# Keratinocyte-neutrophil interactions revealed as targetable drivers of sustained inflammation in Sweet syndrome

**DOI:** 10.1172/JCI198494

**Published:** 2025-10-15

**Authors:** Umi Tahara, Masayuki Amagai

**Affiliations:** 1 Department of Dermatology, Keio University School of Medicine, Tokyo, Japan.; 2 Laboratory for Skin Homeostasis, RIKEN Center for Integrative Medical Sciences, Yokohama, Japan.

## Abstract

Neutrophils are key drivers of inflammation in Sweet syndrome (SS), a rare inflammatory skin disorder, but how they remain persistently activated in SS skin lesions has been unclear. In this issue of the *JCI*, Huang, Sati, and colleagues applied single-cell RNA-Seq and immunofluorescence to identify a subset of neutrophils in SS skin that display antigen-presenting cell–like (APC-like) features. The authors showed that when neutrophils interacted with keratinocytes, their lifespan was markedly extended, and they expressed MHC class II via activation of the serum amyloid A1/formyl peptide receptor 2 (SAA1/FPR2) signaling pathway. This, in turn, enabled T cell activation and sustained self-perpetuating inflammatory loops. These findings reveal a previously unrecognized keratinocyte-neutrophil circuit in SS and point to the SAA1/FPR2 axis as a potential target for more precise, mechanism-based therapy.

## Unraveling enigmatic roles of neutrophils in Sweet syndrome

**Figure 1:**
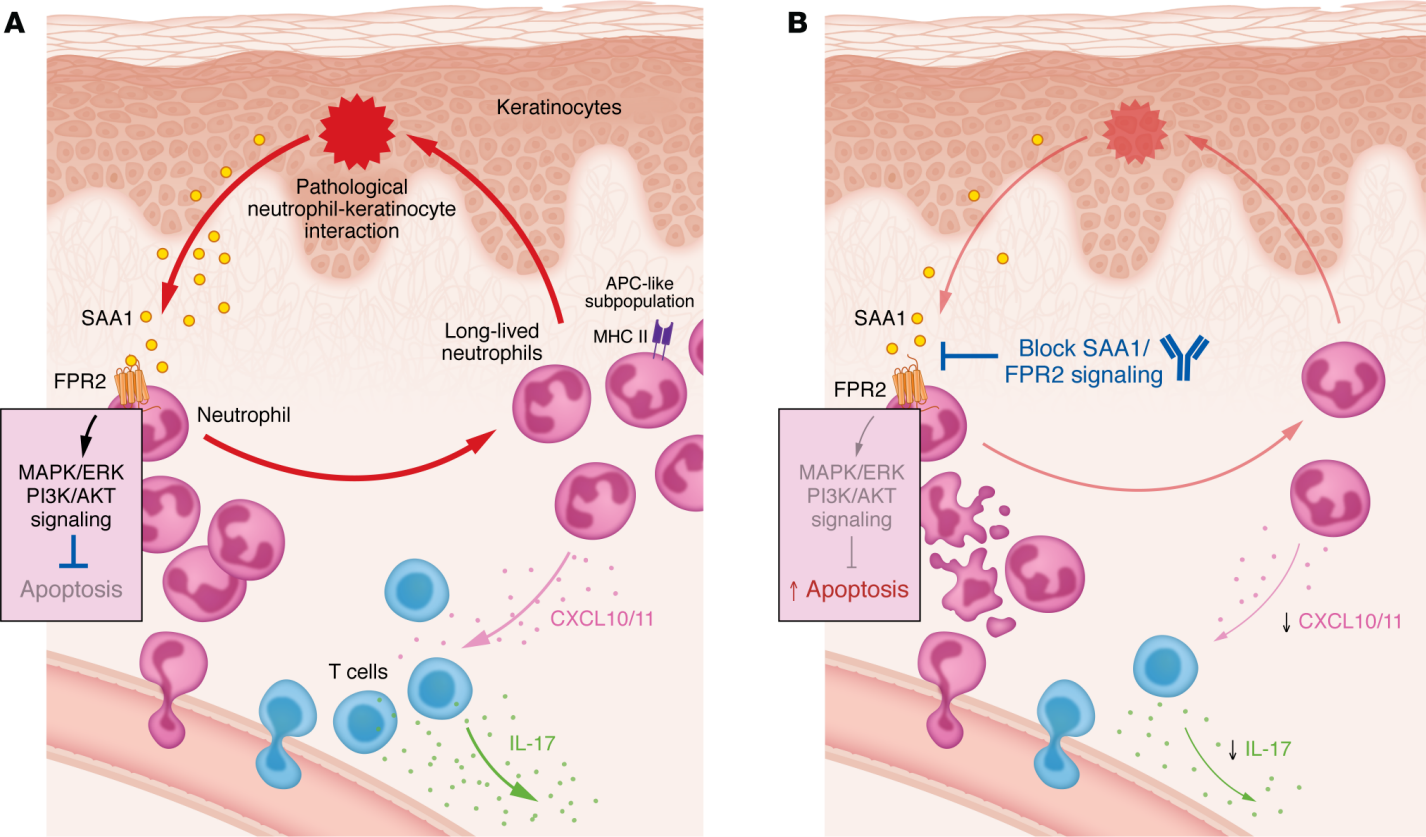
SAA1/FPR2 signaling prolongs neutrophil survival and provides a therapeutic target in SS. (**A**) The keratinocyte-neutrophil interaction that occurs in SS skin, as reported by Huang, Sati, and colleagues (10). Keratinocytes, the predominant epidermal cell type, upregulate SAA1 in response to neutrophil interaction. Keratinocyte-derived SAA1 binds FPR2 on neutrophils, activating MAPK/ERK and PI3K/AKT signaling to inhibit apoptosis and extend neutrophil lifespan. This interaction maintains a pool of long-lived neutrophils, including a subset with APC-like features such as MHC II expression. Lesional neutrophils also produce CXCL10 and CXCL11, recruiting T cells that generate IL-17, linking neutrophil activity to Th17-type inflammation. (**B**) The effect of SAA1/FPR2 blockade. Blocking SAA1/FPR2 signaling with neutralizing antibodies promotes neutrophil apoptosis, thereby reducing the neutrophils’ survival. This axis represents a potential point of therapeutic intervention for SS.

Sweet syndrome (SS), first described in 1964 as “acute febrile neutrophilic dermatosis” (1), is a rare inflammatory skin disorder marked by the abrupt onset of fever, leukocytosis, and tender erythematous plaques or nodules with dense dermal neutrophilic infiltrates (Figure 1). Although SS can be triggered by diverse factors — including infections, autoimmune diseases, malignancies, and certain medications — evidence suggests that a shared pathogenic mechanism operates across subtypes (2). A central feature of SS is innate immune dysregulation. Patients often show elevated levels of granulocyte-CSF (G-CSF), IL-1β, and other proinflammatory cytokines in serum and lesional skin (3–8). These mediators drive neutrophil recruitment into the dermis, aided by upregulation of chemoattractants such as CXCL1, CXCL2, and IL-8, as well as adhesion molecules including l-selectin, FAS, and Siglec-5/9 (9). While these findings underscore the pivotal role of neutrophils, exactly how they initiate and sustain inflammation has been unclear.

A 2023 study from the Leung group described a chronic, treatment-refractory SS patient carrying a neutrophil-specific *PIK3R1* mutation that enhanced IL-1β–driven migration and responded dramatically to IL-1 receptor blockade (7). This work provided direct evidence that neutrophils can act as upstream disease initiators rather than mere downstream effectors. However, that work relied on bulk transcriptomics and monoculture assays with neutrophil-like cell lines — approaches that do not recapitulate the cellular diversity or intercellular signaling within SS lesions. To address this gap, in the current *JCI* issue, Huang, Sati, and colleagues from the same research group combined single-cell RNA-Seq (scRNA-Seq) with in vitro coculture assays to dissect the SS lesional microenvironment (10). Their results support a model in which neutrophils sustain chronic inflammation through reciprocal interactions with keratinocytes, the predominant epidermal cell type. Central to this circuit is the serum amyloid A1/formyl peptide receptor 2 (SAA1/FPR2) signaling axis, which was identified as a potential therapeutic target (Figure 1).

## Antigen-presenting cell–like neutrophils: a tissue-adapted inflammatory phenotype

Neutrophils are notoriously difficult to analyze by scRNA-Seq because of their fragility and low RNA content as well as the presence of proteases and nucleases that can degrade nucleic acids (11). Huang, Sati, and colleagues optimized tissue preparation to reduce cellular stress, enabling reliable capture and transcriptional profiling of neutrophils from skin biopsies.

A key discovery from this analysis was a subset of neutrophils in SS lesions that expressed MHC class II (MHC II) molecules — an unusual feature for this cell type. This aligns with growing evidence that, under certain inflammatory conditions, human neutrophils can acquire characteristics reminiscent of antigen-presenting cells (APCs) (12). Such MHC class II–positive neutrophils have been described in rheumatoid arthritis, allergy, and certain cancers, particularly at inflamed tissue sites (13–15). Whether these “APC-like” neutrophils function as true antigen presenters remains debated. In many settings, MHC class II expression may signal an activated or highly interactive state rather than full antigen-processing capability (12). In SS, this APC-like phenotype was absent in circulating neutrophils from patients, suggesting that local cues within the skin microenvironment reprogram infiltrating neutrophils into a distinct, tissue-adapted state. Even if the antigen presentation capacity of these APC-like neutrophils is limited, their presence underscores the remarkable plasticity of neutrophils and their ability to influence both innate and adaptive immunity in chronic skin inflammation.

## Keratinocyte-neutrophil crosstalk sustains neutrophil survival and inflammation

One of the study’s most striking findings is a self-perpetuating inflammatory loop between keratinocytes and neutrophils. scRNA-Seq and immunofluorescence analyses showed that keratinocytes in SS lesions upregulated SAA1, a major acute-phase reactant protein (16). In vitro, direct contact between keratinocytes and neutrophils boosted SAA1 secretion, which then bound to FPR2 on neutrophils. This signaling promoted neutrophil survival, although additional keratinocyte-derived factors appeared to be required to induce the APC-like phenotype (Figure 1). The observed survival effect is consistent with prior evidence that SAA1 can delay neutrophil apoptosis via FPR2-mediated MAPK/ERK and PI3K/AKT signaling (17). This keratinocyte-neutrophil circuit illustrates how local feedback loops can sustain inflammation and amplify responses triggered by upstream insults. Building on previous reports of the elevated Th1-related cytokines (e.g., IL-12, IFN-γ, CXCR3) and Th17 pathway components (e.g., IL-17A, IL-17E, IL-17R) in SS lesions (9, 18–21), the authors showed that lesional neutrophils expressed high levels of the T cell–recruiting chemokines CXCL10 and CXCL11. In coculture, long-lived neutrophils induced T cells to produce IL-17, linking neutrophil activity to Th17-type inflammation in SS (Figure 1). While these results outline a compelling mechanistic model, much of the evidence comes from in vitro systems, and confirmation in larger patient cohorts or relevant in vivo models will be important to establish physiological relevance and generalizability.

## Therapeutic implications and future directions

By identifying the SAA1/FPR2 axis as a key driver of keratinocyte-neutrophil crosstalk in SS, Huang, Sati, and colleagues highlight a potential new therapeutic target. In their experiments, dual blockade of SAA1/FPR2 signaling with neutralizing antibodies effectively curtailed the extended lifespan of neutrophils (Figure 1). This represents a notable advance over current treatment options, which remain limited. Systemic corticosteroids, which still are the mainstay, carry serious side effects and are prone to prompting relapses upon tapering or discontinuation. Other agents, such as cyclosporine, dapsone, potassium iodine, and the IL-36 receptor inhibitor spesolimab, have variable efficacy and suboptimal long-term safety (22, 23). Crucially, these approaches are broadly immunosuppressive and do not specifically disrupt the pathogenic circuits sustaining SS. Targeting the SAA1/FPR2 pathway offers the prospect of more precise intervention that spares systemic immunity.

The keratinocyte-neutrophil loop, however, is unlikely to act in isolation. Recent work shows that fibroblasts in SS lesional dermis can also promote neutrophil recruitment via IFN-stimulated chemokines and adhesion molecules (24). This broader view of the tissue microenvironment, as an active network involving immune cells and structural cells alike, should inform future therapeutic strategies. Advances in single-cell transcriptomics now make it possible to capture and functionally profile neutrophils from skin biopsies. Given that skin is accessible for minimally invasive sampling, this approach provides a valuable platform for mapping immune cell–structural cell interactions at high resolution and could be adapted to less accessible organs.

In summary, the demonstration of keratinocyte-neutrophil inflammatory loops in the pathogenesis of SS not only reframes neutrophils as active sustainers of chronic skin inflammation but also lays the groundwork for mechanism-based therapy. Comparative studies will be essential to determine whether the SAA1/FPR2 axis is unique to SS or shared across related disorders, guiding broader therapeutic development.

## Funding support

Japan Society for the Promotion of Science Grant-in-Aid for Scientific Research award JP22H04994.

## Version 1 10/15/2025

Electronic publication
